# Constipation and Some Prominent Remedies for Its Treatment

**Published:** 1888-07

**Authors:** Alexander C. Hermance

**Affiliations:** Rochester, N. Y.


### CONSTIPATION AND SOME OF THE MOST PROMINENTLY INDICATED REMEDIES IN ITS TREATMENT.

The intestinal apparatus should in a healthy individual complete its revolution once in twenty-four hours. During this period, the whole process of digestion should be completed, from the entrance of the food into the stomach to the expulsion of the fæces. Any deviation from this should be regarded as a departure from health. However, in some cases, due to constitutional peculiarities, the bowels do not act more than once or twice a week, and yet the individual apparently enjoys good health. Constipation is a mere symptom, and not a disease, and dependent upon some other derangement of the organism, which must be found and corrected if possible, after which the troublesome costiveness so complained of by the patient will have disappeared. How often is this trouble treated as if it were a disease itself, and with but one symptom, especially by the average old-school physician and by the mongrels, who, through indolence or ignorance, more frequently the latter, prescribe anything that will produce an immediate movement of the bowels, be it what it may, an ounce of *Castor Oil* or a *Warner Liver-pill*, and the means thus used to remove the difficulty too frequently operates to fasten it upon the system. This very distressing trouble arises from a great variety of causes: sedentary habit, close mental application, grief, sorrow, derangement of the stomach and liver from improper food, cathartic medicine, astringents, etc. From whatever cause, we must endeavor to find it, and, as good Hahnemannians, fit our remedy to the individual case according to our acknowledged law. I think I am safe in saying that the most frequent cause of this trouble is the taking of cathartics, ignorantly, perhaps, by the patients themselves, and the prescribing of them scientifically by the aforementioned physicians, the result of which, as I have said, is to increase the difficulty and lay the foundation of incurable diseases, making life a burden to many an individual.

In the treatment of constipation, the habit of the patient is to be first considered. There is no physician who has not often been amused by what patients consider constipation. One thinks himself costive unless he has a movement two or three times a day, while another having but that number a week considers his bowels regular. The habit of going regularly to solicit an evacuation is of much importance in patients that are in the habit of postponing the same. The diet also must be carefully considered, the drinking of strong coffee and tea or improper food being a prominent cause. Let us look for the pathological condition, find the cause, if possible, and prescribe, not upon the "sewer flushing" principle, but according to law of similiars, totality of the symptoms, the single remedy, and the dynamic power of the drug.

To mention all the remedies that may be useful in this trouble would be too much to attempt in a paper of this kind, so I have confined myself to a few of the most frequently indicated ones, giving but the prominent characteristics for the same.

*Æsculus hippocastanum.*

One of the most prominent symptoms of this remedy is the *constant backache.* Prolapsus ani after stool with *backache.* Constant dull *backache.* Soreness, lameness, aching in the *back,* always worse from motion, especially walking (also Bry.). Constant urging with ineffectual effort (also Nux vom.). Large, dark, dry stool, with feeling as if the *rectum* was full of *sticks.* *Hemorrhoids,* with the characteristic *backache.* Feeling of fullness, dryness, and *sticks* in the rectum.

*Alumina.*

*Constipation of nursing children* (also Verat. alb.), inactivity of the rectum, *even soft stool requires much effort to expel.* No desire for nor ability to pass stool until there is a large accumulation. Ailments from lead poisoning (also Opium). Stools hard, knotty, and covered with mucus. [Comp. with Plumb., Opium, and Sil.]

*Amm. muriaticum.*

The fæces are covered with a glairy mucus. *Obstinate and extreme constipation, with much flatus.* Hard, crumbling stool (also Mag. mur.). Stools large and hard, followed by soft stool (also Anac.). *Particularly useful if hemorrhoids occur after suppression of leucorrhœa.*

*Anacardium.*

*Great desire, but with the effort the desire passes away.* Frequent ineffectual urging. The rectum seems powerless, as if plugged.

*Hemorrhoids.* Sensation of a hoop or band around parts. Hemorrhoids with constipation.

*Antimonium crudum.*

Alternate diarrhœa and constipation, especially of old people (also Bry. and Phos.). Stool hard, with difficult expulsion.

*The thick, milky, white coating on the tongue, and gastritis aggravated by sour things,* are prominent characteristics of this remedy.

*Bryonia.*

This remedy and *Nux vom.* are two of the first remedies we think of in this trouble, and for that reason they are too often given in an empirical way. The stool of Bryonia is *hard and dry, as if burnt.* Severe frontal headache, coming on in the morning and gradually increasing until evening (also Spigelia), or headache extending backward and down the shoulders, *all troubles worse from motion. Very irritable, parched lips. Thirst for large quantities of water.* This remedy is frequently indicated in troubles arising from warm changes in weather. This I have often verified.

*Chelidonium.*

*Stool like sheep's dung* (also Plumb., Rut., and Mag. m.). *Constant dull pain under lower and inner angle of right shoulder blade.* Hepatic disease with jaundice.

*Ignatia.*

Constipation from taking cold or riding in a carriage (also Platina). Stitches from anus up rectum, inactivity with anxious desire, prolapsus with every stool (also Podoph., Rhus tox., and Sep.), full of suppressed grief with empty feeling in the stomach, bad effects from the use of tobacco.

*Lycopodium.*

Stool very hard and scant, and passed with great difficulty. Ineffectual urging, especially in the evening. Sensation after stool as if much remained. Much fermentation in abdomen, loud rumbling and gurgling in bowels, red sand in the urine (also Phos., Sil., and Dig.). Acidity and heartburn, with great drowsiness after eating. Aggravation from four to eight P. M. Sense of satiety after eating very little.

*Magnesium mur.*

Large, difficult stool, which crumbles when voided (*also Amm. mur.*) like sheep's dung (*also Plum., Ruta, and Chel.*), covered with blood and mucus. Absence of desire; atony as with the bladder.

*Nitric acid.*

Painless constipation. Stool hard, dry, and scant; head feels as if surrounded by a light band (*also Anacardium, Merc., and Sulph.*). Sour eructations; sour, bitter taste after eating (also Nux). *Strong-smelling urine, like horses'*; fissures in anus; tearing and spasmodic symptoms during stool; lancinating after, even after soft stool.

*Nux vomica.*

This remedy is so thoroughly known I can say but little about it. Large, hard stool, passed with difficulty, frequent urging without effect (also Bry. and Lyc.). Victims of drugs; high livers; blind and bleeding piles; *sensation of stone or lump of lead in stomach, frequent eructations of sour or bitter fluids;* persons of sedentary habit and pregnant women (also Bry., Lyc., and Sep.). Frequent desire; passing small quantities; feeling as if not done; bad effects from coffee, rich food, late hours; head feels distended.

*Opium.*

A most excellent remedy when indicated. *Torpidity of the bowels* after chronic diarrhœa or abuse of cathartics (also Nux); stool nothing but *small, hard, black balls* (also Plumb.); constipation from fright or fear (Gels. has diarrhœa from same); costive for weeks with loss of appetite; paralysis of intestines from lead poisoning.

*Phosphorus.*

Fæces long, slender, narrow, tough like a dog's, very difficult to expel (also Caust.). Alternate diarrhœa and constipation of old people (also Bry. and Ant. c.).

*Platinum.*

Stool scant, like putty, *sticking to the anus like soft clay;* constipation while traveling; low-spirited and very nervous; after an evacuation sensation of weakness or chilliness in the abdomen; suitable after lead poisoning (also Alumina and Opium).

*Plumbum.*

Constipation with violent colic; stool composed of little, hard, black-brown balls (also Opium) like sheep's dung (also Chel. and Ruta).

*Pulsatilla.*

Constipation consequent upon eating *rich, greasy food.* Alternate diarrhœa and constipation. Adapted to females or persons of mild, gentle, yielding disposition.

*Ruta grav.*

Constipation following mechanical injuries; great difficulty of voiding stool on account of protrusion of the rectum; frequent urging with protrusion of the rectum; like sheep's dung.

*Sepia.*

*Sense of weight or lump in rectum, not relieved by stool;* especially suited to pregnant women, or females suffering from uterine difficulties; pain in rectum during and long after stool; hard, knotty, insufficient stool covered with blood and mucus (also Mag. mur.); involuntary straining; *yellow saddle across nose.*

*Silicea.*

Difficult expulsion of even soft stool; after much straining it seems to recede into the rectum after being partially expelled; the rectum seems to have no power to expel it; constipation of females particularly before and during menstruation, also of infants and scrofulous children.

*Sulphur.*

Constipation with convulsions, alternating with diarrhœa. First efforts very painful, compelling one to desist. Constant heat on vertex; frequent faint spells.

*Sulphuric acid.*

*Much debility, with a tremulous sensation over the body without trembling;* knotty stools streaked with blood and very fetid.

*Thuja.*

Ineffectual urging with eructations; *hard balls;* obstinate constipation from inactivity or intussusception; violent pain in rectum during stool.

*Veratrum alb.*

Chronic constipation of infants (also Alum and Sil.); inactivity, rectum seems paralyzed; much straining, *with cold perspiration;* great exhaustion and fainting after stool.

ALEX. C. HERMANCE, M. D.

ROCHESTER, N. Y., April 9th, 1888.

